# Joint analysis of m^6^A and mRNA expression profiles in the testes of idiopathic nonobstructive azoospermia patients

**DOI:** 10.3389/fendo.2022.1063929

**Published:** 2022-12-15

**Authors:** Qiuqin Tang, Wei Wu, Yiwen Lu, Yijie Zhou, Wangfei Wu, Jinhui Li, Lianjun Pan, Xiufeng Ling, Feng Pan

**Affiliations:** ^1^ Department of Obstetrics, Women’s Hospital of Nanjing Medical University, Nanjing Maternity and Child Health Care Hospital, Nanjing, China; ^2^ State Key Laboratory of Reproductive Medicine, Institute of Toxicology, Nanjing Medical University, Nanjing, China; ^3^ Key Laboratory of Modern Toxicology of Ministry of Education, School of Public Health, Nanjing Medical University, Nanjing, China; ^4^ Department of Pathology, Women’s Hospital of Nanjing Medical University, Nanjing Maternity and Child Health Care Hospital, Nanjing, China; ^5^ Department of Urology, Stanford University Medical Center, Stanford, CA, United States; ^6^ Department of Andrology, Women’s Hospital of Nanjing Medical University, Nanjing Maternity and Child Health Care Hospital, Nanjing, China; ^7^ Department of Reproductive Medicine, Women’s Hospital of Nanjing Medical University, Nanjing Maternity and Child Health Care Hospital, Nanjing, China

**Keywords:** m^6^A, methylation, azoospermia, spermatogenesis, male infertility

## Abstract

**Background:**

Growing evidence has indicated that epigenetic factors might be associated with the pathophysiology of idiopathic nonobstructive azoospermia (iNOA). As the most common RNA modification, N6-methyladenosine (m^6^A) methylation has recently attracted more attention in the regulation of spermatogenesis; however, its role in the mechanisms of iNOA is still unclear.

**Objective:**

To determine the differential expression of mRNA and m^6^A methylation status in the testes of iNOA patients.

**Methods:**

Testes tissues from diagnosed iNOA and controlled obstructive azoospermia (OA) patients were collected and grouped according to the histological examinations. Total RNA was isolated and quantified by an m^6^A RNA Methylation Quantification Kit. The expression level of mRNAs was detected by qRT-PCR analysis. Differentially expressed m^6^A genes were analyzed using the human ArrayStar m^6^A epitranscriptomic microarray, and bioinformatics analyses were applied.

**Results:**

A total of 36 iNOA and 8 controlled patients were included. The global expression of m^6^A in the iNOA group was significantly decreased. A dosage relationship was observed between the m^6^A decline and the degree of impaired spermatogenesis, with the successive process of normal spermatogeneis, hypospermatogenesis (HP), maturation arrest (MA), and Sertoli cell-only syndrome (SO). Four down-expressed genes (*BDNF, TMEM38B, RPL3L, and C22orf42*) displayed significantly lower expression of m^6^A methylation. Additionally, they also showed a gradually down-expressed tendency in the three groups (OA, HP, SO/MA groups). Moreover, m^6^A reader EIF3A was approved to have differential expression through microarrays analysis, which was consistent with the result from the qRT-PCR test.

**Conclusions:**

The m^6^A expression was gradually downregulated in the testes tissue from iNOA patients in accordance with the degree of spermatogenic dysfunction. The determined differential expression of mRNA and m^6^A methylation status may represent potentially novel molecular targets for the mechanism study of iNOA in the epigenetic level, which could benefit the understanding of the pathophysiology of iNOA.

## Introduction

Infertility affects approximately 15% of couples of reproductive age worldwide, where male factors account for up to 50% of cases (1). As the most severe condition, nonobstructive azoospermia (NOA) contributes to 10-15% of male infertility, with an incidence rate of 1% in the adult male population (2). There are multiple identified factors that could lead to NOA, including chromosomal abnormalities (Klinefelter syndrome, Y-chromosome microdeletions, etc.), cryptorchidism, and chemotherapy or radiotherapy history. However, collectively, these reasons may account for just 30% of NOA instances (2, 3). With ineffective medical therapy and unpredictable sperm extraction rates, the remaining idiopathic NOA (iNOA) cases whose pathological results include Sertoli cell-only syndrome (SO), maturation arrest (MA), and hypospermatogenesis (HP), are the most challenging and frustrating ones in the clinical practice.

With the advancement of epigenomics, it is becoming increasingly apparent that the mechanisms of iNOA might be accompanied by epigenetic variables (4). Generally, the epigenetic modification includes DNA methylation, histone modifications, and non-coding RNAs (5, 6). Nowadays, RNA modifications have shown a critical role in the epigenetic programming of spermatogenesis, with N^6^-methyladenosine (m^6^A) being the most common (7, 8). With three well-established regulation patterns, namely m^6^A writers, erasers, and readers, m^6^A has shown its extensive function in mRNA splicing and stability, microRNA processing, and so on. In spermatogenesis research, the deletion of m^6^A writers, Mettl3 and Mettl14, could disrupt spermiogenesis through the experiments *in vitro* (9), and the ablation of Mettl3 could severely inhibit meiosis in the germ cells (10).

Furthermore, the m^6^A eraser ALKBH5 had an essential role in correct splicing in the round spermatids phase (11). In addition, the elevated expression of Mettl3 and Mettl14 was identified in the semen of asthenozoospermia patients, indicating the correlation between m^6^A and sperm quality (12). However, it is still unclear how m^6^A affects the spermiogenesis process; to our knowledge, there have been only a few reports so far on the subjects.

In this case-control study, we collected the testes tissues from iNOA patients and controlled obstructive azoospermia (OA) patients. Additionally, the testes tissues from iNOA patients were divided into SO/MA or HP groups, indicating the extent of spermatogenesis damage according to the pathological findings. m^6^A and mRNA expression profiles were then tested among all the groups by applying ArrayStar m^6^A microarrays, and verified by qRT-PCR. Bioinformatic analysis was also employed to predict the differentially expressed genes involved in the regulation of m^6^A methylation for spermatogenesis in iNOA.

## Materials and methods

### Testes tissue collection

Approved by the Ethics Committee of Women’s Hospital of Nanjing Medical University, Nanjing Maternity and Child Health Care Hospital (2019KY-027), this study was conducted in the reproductive medical center of Nanjing Maternity and Child Health Care Hospital from November 2019 to February 2021. For the case group, patients with male infertility who had been diagnosed with azoospermia were enrolled. The semen analysis, with a complete lack of sperm in the ejaculate at least twice, combined with hormonal evaluation, was used to determine the diagnosis of iNOA. The exclusion criteria include 1) obstructive azoospermia (OA), which diagnosis was based on the physical examination, sex hormone testing, and transrectal ultrasound performed by a skilled urologist; 2) NOA with genetic causes (abnormal karyotype, pathogenic Y-chromosomal microdeletions, etc.); 3) NOA with a detectable disorder affecting hypothalamic-pituitary-gonadal axis based on hormonal evaluation and imaging diagnoses (hypogonadotropic hypogonadism, hyperprolactinemia); 4) NOA caused by secondary testicular failures, such as cryptorchidism and iatrogenic factors (chemotherapy, radiotherapy). Besides, the OA patients who chose ICSI with testicular sperm extraction (TESE) were included in the control group, and who chose to undertake anastomosis were excluded in this study. All the participants’ testes tissues were collected using the residual tissues after the TESE operation with written informed consent.

### Histological analysis

All collected testes tissues were fixed in 4% Paraformaldehyde (PFA) for 24 hours and then embedded with paraffin after dehydration. Next, samples were cut into 4-micron sections and stained with hematoxylin and eosin (H&E) using a fully automatic H&E staining machine and Dako CoverStainer (Agilent Technologies, Inc., USA). The improved Mayer hematoxylin (CS700, Dako) was used in the process. Finally, the stained slides were evaluated, and the images were analyzed under a light microscope (Eclipse 80i, Nikon, Japan) at 100× to 400× magnifications.

### RNA extraction and m^6^A quantification of the overall m^6^A levels

Total RNA was isolated from each testicular sample using TRIzol reagent (Invitrogen; Thermo Fisher Scientific, Inc.). The concentration and purity of total RNA samples were determined using NanoDrop 2000 (Thermo Fisher Scientific, Inc.). The total RNA samples were stored at -80˚C for subsequent experiments. The m^6^A quantification was conducted using the m^6^A RNA Methylation Quantification Kit (Epigentek) following the manufacturer’s protocol. Briefly, 200 ng total RNA sample was added to each test well, followed by the addition of capture antibody and test antibody in turn, and then the reaction was stopped with stop solution. Finally, the absorbance at 450 nm of the test wells was detected by a microplate reader to calculate the percentage of m^6^A in total RNA.

### Analysis of related mRNA using qRT-PCR

The reverse transcription step to cDNA was performed using the PrimeScript RT reagent Kit (Takara, Tokyo, Japan), and qRT-PCR analysis was conducted by SYBR^®^ Premix Ex Taq™ on the LightCycler 480 II real-time fluorescent quantitative PCR system (Roche Diagnostics). The 2^-ΔCt^ method was used to calculate the relative expression level of related mRNAs. GAPDH was used as an internal control for mRNA quantification. All the reactions were run in duplicate. The primers used for qRT-PCR in this study were verified by previous published papers which sequences were listed in **Table 1**.

**Table 1:** Sequences of primers for mRNAs qRT-PCR analyses.

Gene symbol	Primers	Sequences (5’-3’)
*METTL3*	Forward	AGCTGCACTTCAGACGAAT
	Reverse	GGAATCACCTCCGACACTC
*METTL14*	Forward	AGAAACTTGCAGGGCTTCCT
	Reverse	TCTTCTTCATATGGCAAATTTTCTT
*WTAP*	Forward	GGCGAAGTGTCGAATGCT
	Reverse	CCAACTGCTGGCGTGTCT
*VIRMA*	Forward	CGATAACTTGATGACCCCAGAA
	Reverse	ATAACGGCAAGATTCCATTTC
*RBM15*	Forward	TCCCACCTTGTGAGTTCTCC
	Reverse	GTCAGCGCCAAGTTTTCTCT
*RBM15B*	Forward	CGAGAGTTTGACCGCTTTGGG
	Reverse	CCGAGTCTCCTCTGCTTTGGC
*ZC3H13*	Forward	AGAAAAGAGAGACAAGCCAAGGT
	Reverse	GAGAGGCAGAGCGTCGTAAAG
*METTL16*	Forward	TTCCTCGCAACAGAAGTGGA
	Reverse	GTCTTCTGTGGCACTTTCACC
*FTO*	Forward	GGGTTCATCCTACAACGG
	Reverse	CTCTTCAGGGCCTTCAC
*ALKBH5*	Forward	CCGAGGGCTTCGTCAACA
	Reverse	CGACACCCGAATAGGCTTGA
*YTHDF1*	Forward	CTCAGCATGGGGGACAAGTG
	Reverse	GAGGAGCTGACGTCCCCAAT
*YTHDF2*	Forward	GGCAGCACTGAAGTTGGG
	Reverse	CTATTGGAAGCCACGATGTTA
*YTHDF3*	Forward	CTACTTTCAAGCATACCACCTCAA
	Reverse	GCATTTCCAGAGTCTACATCGTTA
*YTHDC1*	Forward	CCACACCATCCTTACTATCAGCA
	Reverse	CTCTTTCACGGGGTCTACTTCTC
*YTHDC2*	Forward	GGAGCCAATGTCCATAGTAAAGC
	Reverse	ACTTCCATTTGTTTGAACCAGAG
*IGF2BP1*	Forward	GTCCCTCTCCCGTGTAGGTTTC
	Reverse	AGTTAGCGTCCCCTTCCCAGTG
*IGF2BP2*	Forward	GTGGCAAGACCGTGAACGAACT
	Reverse	TGCTCCTGCTGCTTCACCTGTT
*IGF2BP3*	Forward	AGTTGTTGTCCCTCGTGACC
	Reverse	GTCCACTTTGCAGAGCCTTC
*FMR1*	Forward	AAAAGAGCCTTGCTGTTGGTGGT
	Reverse	TTGTGGCAGGTTTGTTGGGATTA
*EIF3A*	Forward	TCAAGTCGCCGGACGATA
	Reverse	CCTGTCATCAGCACGTCTCCA
*GAPDH*	Forward	GGATTTGGTCGTATTGGG
	Reverse	CTGGAAGATGGTGATGGGATT

### ArrayStar m^6^A epitranscriptomic microarray analysis

The sample preparation and microarray hybridization were performed according to Arraystar’s standard protocols. Briefly, the total RNAs were immunoprecipitated with anti-m^6^A antibody. Next, the modified RNAs “IP” and the unmodified RNAs “Sup” were labeled with Cy5 and Cy3 using Arraystar Super RNA Labeling Kit. The synthesized cRNAs were then hybridized onto Arraystar Human mRNA&lncRNA Epitranscriptomic Microarray (8×60K, Arraystar). Then, the arrays were scanned using the Agilent Scanner G2505C system.

Agilent Feature Extraction software (version 11.0.1.1) was used to analyze microarray data. First, raw intensities of IP and Sup were normalized and log2-transformed. After Spike-in normalization, the probe signals having Present (P) or Marginal (M) QC flags in at least 3 out of 9 samples were retained as “All Targets Value” for further analyses. Then it was followed by the following procedures: 1) the “m^6^A methylation level” was calculated for the percentage of modification based on the IP and Sup normalized intensities; 2) the “m^6^A quantity” was calculated for the m^6^A methylation amount based on the IP normalized intensities; 3) the “expression level” was calculated based on the total of IP and Sup normalized intensities of RNA. Differentially m^6^A-methylated RNAs or RNAs expression among the three groups were compared by calculating the fold change (FC) with a cutoff of 2-fold (*P* < 0.05). Finally, the clustering analysis heatmaps and the Venn diagram were performed to show the distinguishable m^6^A methylation, m^6^A quantity, and mRNA expression among the three groups.

### Bioinformatic analysis

Gene Ontology (GO) terms, which encompass molecular function, cellular structure, and cellular processes, and Kyoto Encyclopedia of Genes and Genomes (KEGG) pathways enrichment analyses were performed using DAVID (https://david.ncifcrf.gov/). To assess the importance of genes whose m^6^A methylation level was significantly downregulated in the iNOA group, we established an evaluating system using five pieces of evidence that has hierarchical significance (i to v), based on the tendency and cooperativity in the scales of m^6^A methylation, m^6^A quantity, and mRNA expression for differentially expressed genes.

### Statistical analysis

Data were expressed as mean ± SEM. Mann-Whitney U test was used for continuous variables and the Chi-square test for categorical variables (GraphPad software, San Diego, California). One-way ANOVA was used for multiple group comparisons. A *P*-value <0.05 was considered statistically significant (*P < 0.05).

## Results

The testes tissues from 36 iNOA and 8 OA patients were collected in this study. And their demographic characteristics are listed in **Table 2**. The histological results included 7 SO, 17 MA, and 12 HP in iNOA patients. Among them, testes tissues from 30 iNOA patients with 6 SO, 15 MA, and 9 HP cases, and 5 randomly selected OA patients were used for the m^6^A quantification experiment. The tissue samples from the remaining 6 iNOA containing 1 SO, 2 MA, and 3 HP, as well as 3 OA patients were used to perform the ArrayStar m^6^A microarrays. No significant differences could be seen in the comparison of age and BMI in different groups, while the smoking and alcohol drinking status of OA groups were significantly lower (*P* < 0.05). In addition, the LH and FSH values were lower in OA and HP groups when compared with that in the SO or MA groups (*P* < 0.05). Also, the volumes of testes in OA groups were significantly more prominent than in other groups (*P* < 0.05).

**Table 2:** Baseline characteristics, gonadal hormone concentrations, testes volumes and the success testicular sperm extraction rates of all the patients.

	SO	MA	HP	OA
**Age (years)**	27.7 ± 2.7	29.5 ± 3.3	30.5 ± 5.0	29.0 ± 2.4
**BMI (kg/m^2^)**	25.2 ± 2.6	26.7 ± 9.1	23.9 ± 3.5	26.8 ± 9.3
**Alcohol drinking, n (%)**	2 (28.6)	5 (29.4)	4 (33.3)	1 (12.5)*
**Passive smoking, n (%)**	4 (57.1)	9 (52.9)	8 (66.7)	3 (37.5)*
**LH (mIU/mL)**	8.7 ± 2.1	6.1 ± 3.5	4.8 ± 4.5^#^	4.0 ± 1.7^#^
**FSH (mIU/mL)**	22.0 ± 8.7	16.8 ± 7.5	7.2 ± 4.3^#^	5.7 ± 4.3^#^
**E2 (pg/mL)**	36.4 ± 1.5	36.5 ± 1.6	32.9 ± 1.6	34.1 ± 1.5
**T (ng/mL)**	3.6 ± 0.9	3.2 ± 0.8	3.5 ± 1.3	4.1 ± 1.4
**Testis volume (mL)**	11.9 ± 0.4	11.4 ± 2.6	12.4 ± 2.4	15.0 ± 2.5*
**Success testicular sperm extraction, n (%)**	0 (0)	6 (35.3)	9 (75)	8 (100)

SO, Sertoli cell-only syndrome; MA, maturation arrest; HP, hypospermatiogenesis; OA, obstructive azoospermia; BMI, Body Mass Index; LH, Luteinizing hormone; FSH, Follicle-stimulating hormone; E2, Estradiol; T, Testosterone. Mann-Whitney U test was used for continuous variables and Chi-square test for categorical variables. *Compared with other three NOA groups, P<0.05. ^#^Compared with SO or MA groups, P<0.05.

### The expression of m^6^A was downregulated in iNOA patients’ testes

**Figure 1:**
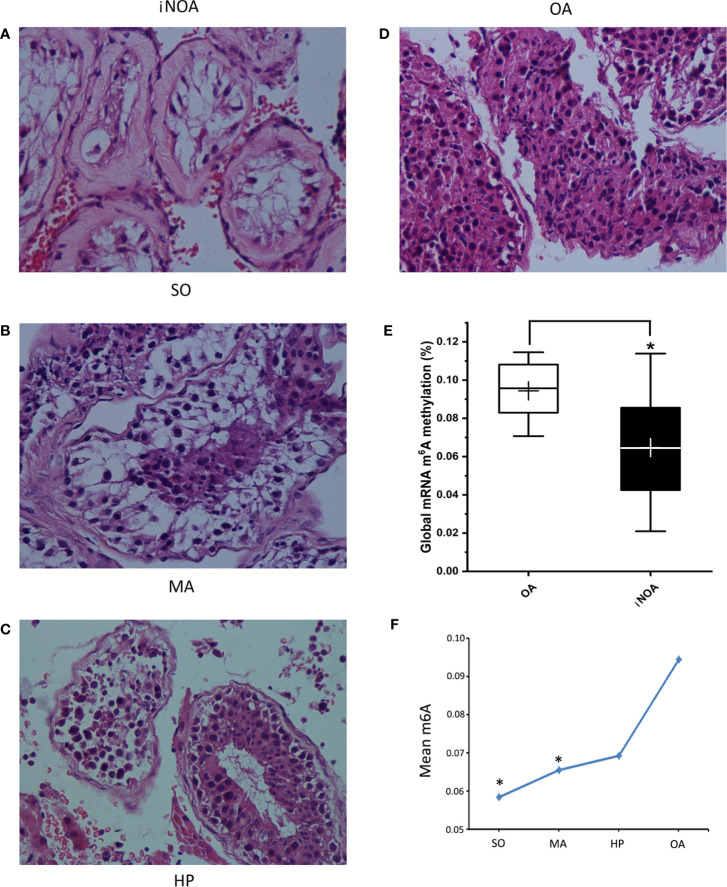
The iNOA group was divided into three groups according to the pathological types, namely the SO group **(A)**, the MA group **(B)**, and the HP group **(C)**. **(D)** shows the histology of OA. All the four representative images are 400× magnifications using H&E staining. **(E)** shows the significantly decreased global expression of m^6^A in testes tissues from iNOA groups. **(F)** shows the a dosage relationship between the m^6^A expression decline and the degree of impaired spermatogenesis. SO: Sertoli cell-only syndrome, MA: maturation arrest, HP: hypospermatogenesis, OA: obstructive azoospermia. *P < 0.05 compared with OA group.

The global expression of m^6^A in testes from iNOA patients was significantly decreased compared to that in the OA group. After dividing the iNOA group into three subgroups according to the pathological types, it was discovered that the m^6^A expression gradually declined from OA to HP, MA, and SO groups, with a dosage relationship observed between the decline and the degree of spermatogenic dysfunction (**Figure 1**).

### iNOA is associated with the alterations of mRNA expression and their m^6^A methylation status

**Figure 2:**
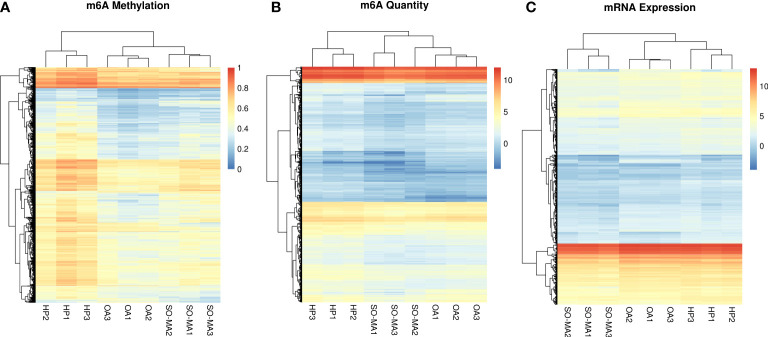
The clustering analysis heatmaps of m^6^A methylation **(A)**, m^6^A quantity **(B)**, and mRNA expression **(C)** in the SO/MA, HP, and OA groups.

The expression profiles of mRNA and their m^6^A methylation status in the SO/MA, HP, and OA groups were identified by the ArrayStar m^6^A microarrays. The three heatmaps showed the clustering analysis of m^6^A methylation, m^6^A quantity, and mRNA expression in the three groups (**Figure 2**).

**Figure 3:**
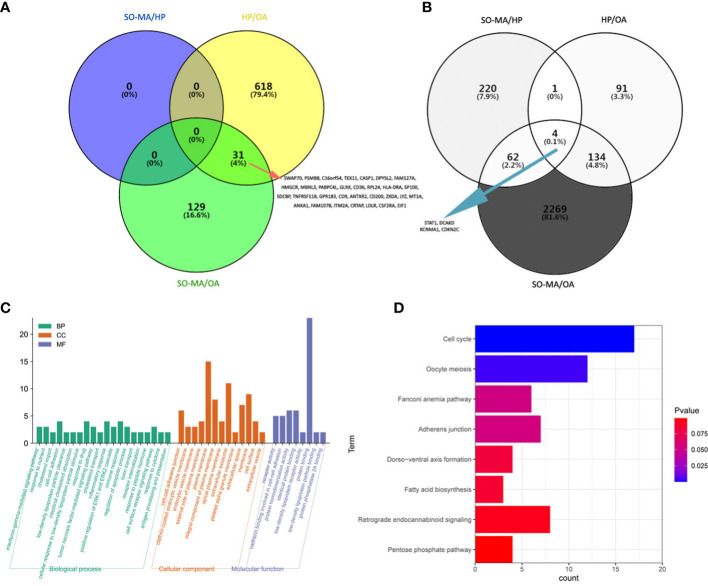
Bioinformatics analysis of differentially expressed genes and m^6^A methylation status in the three groups (the SO/MA, HP, and OA groups). **(A)** The Venn diagram of up-regulated mRNA gene in three groups, and the intersection area included 31 listed genes. **(B)** The Venn diagram of down-expressed mRNA gene in three groups, and the intersection area included four listed genes. **(C)** GO analysis of the 31 up-expressed and 4 down-expressed genes. **(D)** KEGG pathway analysis of 141 genes with significantly lower m^6^A methylation quantity both in the SO/MA and HP groups when compared to that in the OA group.

As a result, there are 160 significantly up-expressed genes in the SO/MA group and 649 in the HP group compared to the OA group. The intersection of the two results found 31 up-expressed genes (**Figure 3A**). Meanwhile, 2469 significantly down-expressed genes from the SO/MA group and 230 ones from the HP group were identified compared to the OA group. Moreover, 287 significantly down-expressed genes were placed in the SO/MA group compared to the HP group. The intersection of the three results highlighted 4 down-expressed genes, which are listed in **Figure 3B**.

Furthermore, the Gene Ontology (GO) analysis was performed to investigate the potential functions of the 31 up-expressed and 4 down-expressed genes. As **Figure 3C** showed, the most significant GO terms related to molecular function and cellular components were protein binding, plasma membrane, and extracellular exosome. We mainly focused on the hypomethylated genes when comparing the difference in gene m^6^A methylation level or m^6^A quantity in three groups. As a result, 9 genes with significantly lower m^6^A methylation levels in the SO/MA group and 7 in the HP group were found compared to the OA group. In addition, there were 1937 genes with significantly lower m^6^A methylation quantity in the SO/MA group and 309 in the HP group compared to the OA group. The intersection of these two results included 141 genes, and their KEGG pathway analysis result was shown in **Figure 3D**. The most enriched KEGG pathway was the cell cycle.

To predict the genes with m^6^A methylation status alteration potentially involved in the mechanisms of iNOA, we further compared the genes with significantly decreased m^6^A methylation levels from the SO/MA and HP groups to the OA group. From **Table 3**, four genes (*BDNF, TMEM38B, RPL3L, and C22orf42*) displayed significantly lower m^6^A methylation status associated with decreased gene expression, which was accompanied by the gradual down-expressed tendency in the three groups, reflecting the degree of spermatogenic dysfunction.

**Table 3:** To predict the importance of differentially expressed genes with significantly downregulated m^6^A Methylation levels in iNOA group using a 5-degree evidence evaluating system.

Gene Symbol	m^6^A Methylation Level (iNOA)	m^6^A Methylation Level (OA)	m^6^A Methylation Quantity (iNOA/OA)	Gene Expression Fold Change (HP/OA)	Gene Expression Fold Change (SO-MA/OA)	Significant Mechanism of Gene m^6^A Methylation in iNOA (Evidence)
**MITF**	6.80%	19.40%	Up	↑↑*	↑	No (i)
**PUM1**	6.80%	18.40%	Up	↑↑*	↑	No (i)
**NPPB**	35.10%	78.50%	Up*	↑	↑↑*	No (ii)
**CLEC7A**	32.30%	66.00%	Down	↓	↑	No (iii)
**GRB10**	20.10%	40.40%	Down	↑	↓	No (iii)
**VSTM5**	20.60%	55.70%	Down	↑	↓	No (iii)
**ARF1**	19.60%	41.20%	Down	↑	↓	No (iii)
**CCDC61**	15.70%	34.30%	Down	↑*	↑	No (iii)
**CTXN2**	21.80%	50.80%	Down*	↓*	↓↓	Yes (iv)
**ANKRD60**	28.50%	64.10%	Down*	↓↓*	↓↓	Yes (iv)
**BDNF**	32.70%	67.50%	Down*	↓	↓*	Yes (v)
**TMEM38B**	14.40%	36.20%	Down*	↓	↓↓*	Yes (v)
**RPL3L**	27.20%	55.00%	Down*	↓	↓*	Yes (v)
**C22orf42**	35.60%	72.60%	Down*	↓↓	↓↓*	Yes (v)

*With statistical significance (P < 0.05).

Evidence i: Lower percentage of m^6^A methylation level of this gene with increased m^6^A methylation quantity in all groups manifesting that erased m^6^A methylation accompanied with enhanced gene transcription; ii: This gene may be important in NOA for its strengthening transcription, but the m^6^A function is attenuated; iii: The gene expression change is unordered in three groups, and with no statistical significance; iv: Significantly declined m^6^A methylation together with decreased gene transcription, but no statistical significance can be found when the gene expression was compared between the SO-MA group and the OA group; v: Significantly lower m^6^A methylation associated with decreased gene expression, and the tendency is gradually in three groups which reflect the degressive spermatogenesis. ↑-expressed and ↓-expressed.

### An m^6^A reader was upregulated in the testes of iNOA patients

**Figure 4:**
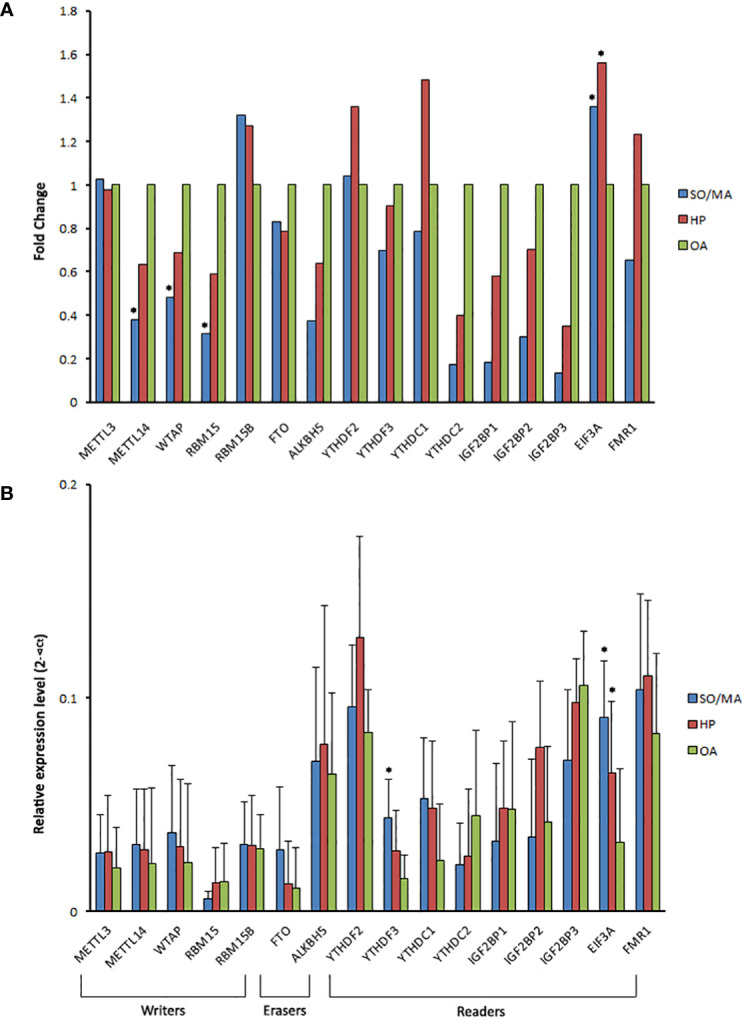
The gene expression of m^6^A related writers, erasers, and readers in the different groups. **(A)** The gene expression fold change of the m^6^A related writers, erasers, and readers in the SO/MA or HP group compared to the OA group *via* the detection of ArrayStar m^6^A microarrays. **(B)** The comparison of m^6^A related regulators’ mRNA expression between the three groups using qRT-PCR. *P < 0.05 compared with the OA group.

Corresponding to the global decline of m^6^A expression in the iNOA group, the majority of m^6^A-related writers’ and readers’ genes were down-expressed in the iNOA groups, especially in the SO/MA group (**Figure 4A**). However, one m^6^A reader, eukaryotic translation initiation factor 3 subunit A (EIF3A), was significantly upregulated in the iNOA group, which was also verified by the qRT-PCR test. As for the significantly declined m^6^A writers (METTL3, WTAP, and RBM15), their ArrayStar m^6^A microarrays results were not testified by the qRT-PCR (**Figure 4B**).

## Discussion

m^6^A plays diverse functions in stem cell pluripotency regulation, posttranscriptional regulation, and mRNA splicing and stability (13, 14). Notably, there is growing evidence in support of its significance in human spermatogenesis (12, 15, 16), which is a highly organized process. A previous clinical study showed that the m^6^A expression in sperm RNA was upregulated in asthenozoospermia (12), in which the researchers illustrated that the abnormal expression of m^6^A might regulate the mRNAs of certain genes related to sperm motility. Being different from the study subject, our study explored the m^6^A expression in the testes tissues of iNOA patients. As is well-known, there is a distinction between the spermatids in testes and the ejected spermatozoa, which experience the capacitation in the epididymis, and have a potential contamination by the somatic cell. Overall, it could be illustrated that dynamic and varied mechanisms participate in the m^6^A regulation in different spermatogenesis processes for male fertility.

It is well known that, spermatogenic and even spermatogonial cells are absent in SO patients. In contrast, the MA patients include the complete MA characterized by the absence of haploid spermatids, and the incomplete MA, in which a few round or later stage spermatids could be found potentially (17). Studies have shown that m^6^A is dynamically present in all mRNAs from different stages of mouse spermatogenic cells, with the peak expression in pachytene spermatocytes and round spermatids (11, 18); thus, the lack of these stages of meiosis with or without spermiogenesis might lead to the decline of m^6^A expression. Consistently, our results also identified the dosage relationship between the m^6^A and different spermatogenesis conditions. The m^6^A expression decreased gradually within OA, HP, MA, and SO gourps, and a significant decline was presented in the MA and SO groups.

It is reported that the m^6^A writers, methyltransferase like 3 (METTL3) and METTL14, likely act critical roles in the spermatogonial stem cells differentiation/proliferation and the spermatids differentiation in the late stages of spermiogenesis (9, 10). METTL14 can form a component with METTL3, as well as strengthen their activities as m^6^A writers (9). Moreover, WTAP, a mammalian splicing factor, was reported to interact with the complex of METTL3- METTL14 and affect m^6^A methylation (19). In our study, METTL14 and WTAP were tested down-expressed based on the ArrayStar m^6^A microarrays’ results, which were not verified by the qRT-PCR. More investigations are warranted in this aspect for further studies.

Based on the findings from Kasowitz SD et al., the YTHDC1 KO mice lacked germ cells and exhibited a SO phenotype, manifesting its necessary regulations in spermatogonial differentiation (20). The YTHDC2 could affect the spermatocyte stage *via* interacting with the meiosis-specific protein MEIOC (21). And YTHDF2 might influence spermatogonia proliferation by affecting the stability of m^6^A-containing transcripts (22). However, these ‘readers’ did not show the significantly differential expressions in iNOA patients’ testes based on our analysis. It might be partially explained by the differences in the spermatogonial stem cells and spermatogenesis stages between humans and rodents (23). By applying the bioinformatic methodology, two newly-emerging fields of study from the same study group revealed that m^6^A methylation-related ALKBH5 and METTL3 were significantly downregulated, and YTHDF3 was upregulated considerably in the iNOA group comparing to the normal group (15, 16). However, their original data were based on Affymetrix Human Gene microarrays’ results, and further verification could provide more evidence on the functions of YTHDF3.

Our study reported that EIF3A was upregulated in the iNOA group, which was not a classic m^6^A reader and seldom studied in spermatogenesis. As the largest subunit of eIF3, eIF3a is a 170-kDa protein consisting of 1382 amino acids, and is a major initiation factor in mRNA translation progress (24). Moreover, eIF3a involves in cell cycle, DNA synthesis and repair regulation, and serves as a negative regulator of cell differentiation in some tissues (24–26). In carcinogenesis studies, eIF3a has been recognized as a proto-oncogene, and may be a potential anticancer drug target in the eIF family (24). Recently, researchers found that eIF3a could involve in fibrosis through regulation of the TGF-β1 signaling pathway (27); the m^6^A reader YTHDF3 could recruit eIF3a to facilitate FOXO3 translation, subsequently initiating autophagy (28). These findings may provide directions for subsequent studies of eIF3a in the mechanisms of iNOA. In the present study, considering that the majority of iNOA spermatogenesis states entire translational repression, we hypothesized that the compensated mechanism might be responsible for the upregulation of EIF3A in the testes of iNOA patients.

Furthermore, four genes (*BDNF, TMEM38B, RPL3L, and C22orf42*) were predicted to be involved in the dysregulation of m^6^A methylation in iNOA mechanisms in the present study. First, Brain-derived neurotrophic factor (BDNF) was detected in human spermatozoa which might influence sperm mitochondrial activity and apoptosis (29, 30). Second, Transmembrane protein 38B (TMEM38B) encodes trimeric intracellular cation channel type B, expressed in most mammalian tissues’ endoplasmic reticulum (31). Finally, Ribosomal protein L3-like gene (RPL3L) is a novel gene located near the PKD1 and TSC2 genes on chromosome 16p13.3, expressed mainly in skeletal muscle and heart tissue (32). It may be involved in the translation initiation. All these genes’ potential functions in iNOA still need further research.

There are some limitations and flaws in this study. First, the testis tissue contains other types of cells besides the spermatogenic cells, such as Leydig cells, Sertoli cells, myoid cells, macrophages, etc. Therefore, the findings might be impacted by the contamination to some extent. Second, the fixation of PFA and HE staining applied in the pathological examination might cause some limitation because the results always displayed shrinkage artifacts between seminiferous tubules and germ cells. Third, the patients’ number was relatively small for that the residual testicular tissue after sperm retrieval in clinic was always not enough for the following RNA experiments. And just because of this, the SO and MA patients were combined into one subgroup in this study. Besides, we considered that the pathology of SO and MA were homologous to some extent, and clinically their sperm retrieval rate were both very low. Nevertheless, more studies with a large population size of iNOA patients are highly desired in the future.

## Conclusions

**Figure 5:**
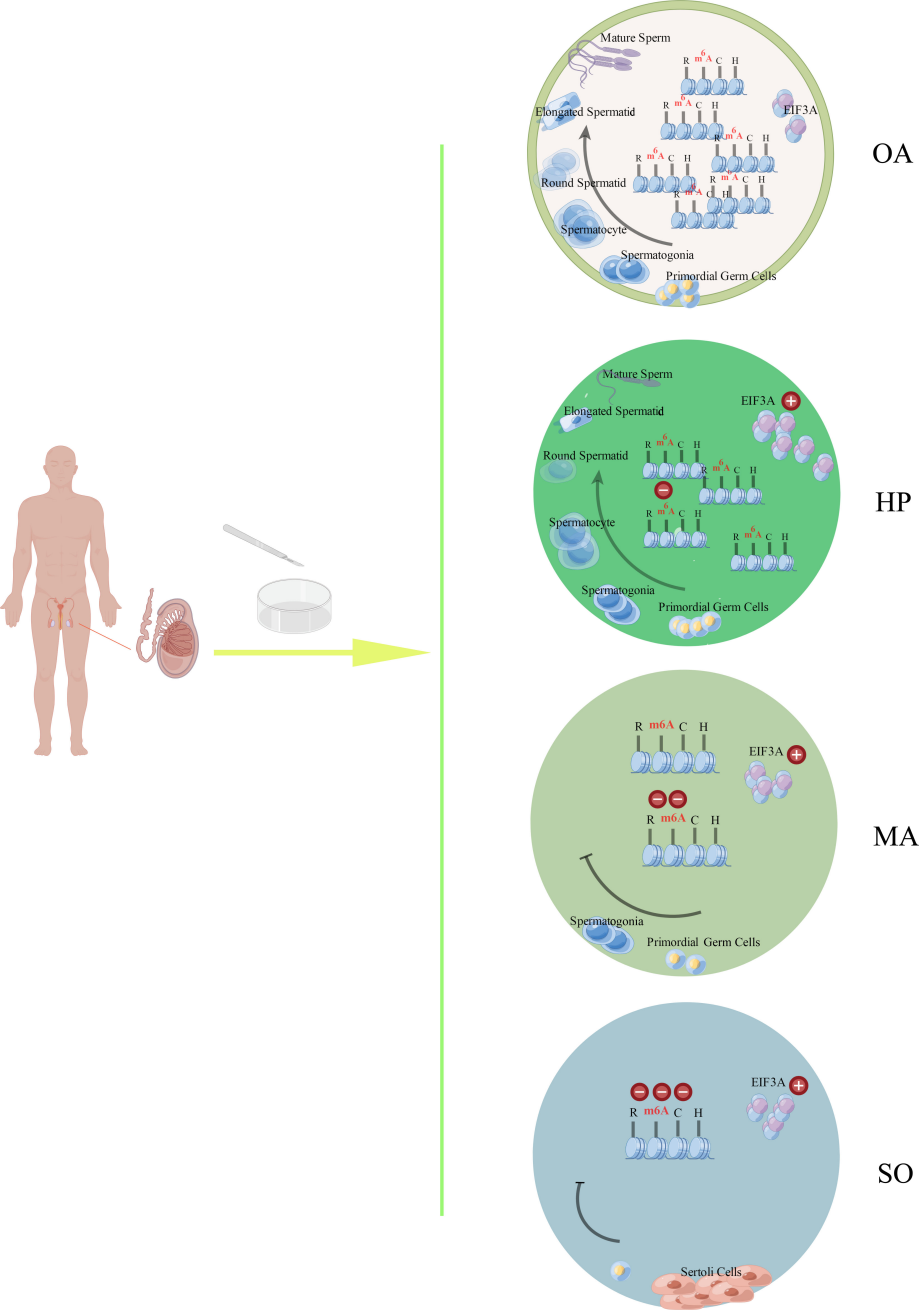
The schematic drawing of the main findings from this study by Figdraw. The m^6^A expression was gradually downregulated in the testes tissues from different groups (OA, HP, MA, and SO groups) in accordance with the degree of spermatogenic dysfunction. As m^6^A reader, EIF3A was testified to be upregulated in the testes from iNOA patients, which include the patients with HP, MA, or SO. OA: obstructive azoospermia (with the successive process of normal spermatogeneis), HP, hypospermatogenesis; MA, maturation arrest; SO, Sertoli cell-only syndrome.

This study, to our knowledge, is a first exploration in the joint expression profiles of m^6^A and mRNA in the testes of iNOA patients. As shown in **Figure 5**, the m^6^A expression was gradually downregulated in the testes tissue from iNOA patients in accordance with the degree of spermatogenic dysfunction. As m^6^A reader, EIF3A was testified to be upregulated in iNOA patients’ testes. iNOA is associated with the alterations of mRNA expression and their m^6^A methylation status. Four genes displayed significantly lower m^6^A methylation status associated with decreased gene expression were hypothesized to be involved in the dysregulation of m^6^A methylation in the iNOA mechanisms. Overall, the determined differential expression of mRNA and m^6^A methylation status may represent potentially novel molecular targets for the mechanism study of iNOA in the epigenetic level.

## Data availability statement

The original contributions presented in the study are included in the article/supplementary materials, further inquiries can be directed to the corresponding author/s.

## Ethics statement

The studies involving human participants were reviewed and approved by The Ethics Committee of Women’s Hospital of Nanjing Medical University, Nanjing Maternity and Child Health Care Hospital. The patients/participants provided their written informed consent to participate in this study.

## Author contributions

Conception and design: QT, WW, and FP Acquisition of data: QT, YL, and YZ. Analysis and interpretation of data: WW, LP, XL, and FP Drafting the manuscript: QT and FP Laboratory testing: YL, YZ, and WFW. Critical revision of the manuscript: WW, LP, XL, FP, and JL. Final approval to be published: all authors. Agreement to be accountable for all aspects: all authors. All authors contributed to the article and approved the submitted version.
